# Hypoxia induced CCL28 promotes angiogenesis in lung adenocarcinoma by targeting CCR3 on endothelial cells

**DOI:** 10.1038/srep27152

**Published:** 2016-06-02

**Authors:** Guichun Huang, Leilei Tao, Sunan Shen, Longbang Chen

**Affiliations:** 1 Medical Oncology Department of Jinling Hospital, Medical School of Nanjing University, Nanjing, China; 2 Medical School of Nanjing University, Nanjing, China

## Abstract

Tumor hypoxia is one of the important features of lung adenocarcinoma. Chemokines might mediate the effects caused by tumor hypoxia. As confirmed in tumor tissue and serum of patients, CC chemokine 28 (CCL28) was the only hypoxia induced chemokine in lung adenocarcinoma cells. CCL28 could promote tube formation, migration and proliferation of endothelial cells. In addition, angiogenesis was promoted by CCL28 in the chick chorioallantoic membrane and matrigel implanted in dorsal back of athymic nude mice (CByJ.Cg-*Foxn1*^*nu*^/J). Tumors formed by lung adenocarcinoma cells with high expression of CCL28 grew faster and had a higher vascular density, whereas tumor formation rate of lung adenocarcinoma cells with CCL28 expression knockdown was quite low and had a lower vascular density. CCR3, receptor of CCL28, was highly expressed in vascular endothelial cells in lung adenocarcinoma when examining by immunohistochemistry. Further signaling pathways in endothelial cells, modulated by CCL28, were analyzed by Phosphorylation Antibody Array. CCL28/CCR3 signaling pathway could bypass that of VEGF/VEGFR on the levels of PI3K-Akt, p38 MAPK and PLC gamma. The effects could be neutralized by antibody against CCR3. In conclusion, CCL28, as a chemokine induced by tumor hypoxia, could promote angiogenesis in lung adenocarcinoma through targeting CCR3 on microvascular endothelial cells.

Angiogenesis, the formation of new blood vessels from preexisting ones, is one of the hallmarks of cancers^1^. The mechanism is very important for cancer progress as it can nourish cancer cells by supplying nutrients and oxygen^2^, so targeting angiogenesis has been proposed as innovative strategy for cancer. Several antiangiogenesis agents have been approved by the FDA for the treatment of lung adenocarcinoma, colorectal cancer, renal cancer and central nervous system tumors^3^. Most of the antiangiogenesis agents were designed to directly or indirectly target the VEGF or its receptors, which has been identified as the key pro-angiogenic factor^4^. However, the clinical outcomes of these drugs were not as effective as predicted. Some reports even argued that anti-angiogenesis therapy might cause the cancer to metastasize, or to rebound after termination of the treatment^5^,^6^.

There are two obstacles for antiangiogenesis therapy, including intrinsic and adaptive resistance to antiangiogensis drugs^7^. However, the mechanism of the drug resistance is not well known so far. Among some identified mechanisms, hypoxia might be a very important one. Under hypoxic conditions, many pro-angiogenic factors, including some chemokines, could be up-regulated and could bypass the classical VEGF-VEGFR pathway under hypoxic condition, which is a common phenomenon in cancer^8^.

Chemokines, a superfamily of structurally homologous heparin-binding cytokine molecules, are critical mediators of neovascularization in many physiologic and pathologic states, such as cancer and inflammation^9^. Structurally, chemokines are grouped into 4 families (designated CC, CXC, C, and CX3C) based on the location of conserved cysteine residues near their amino-terminus^10^. Chemokine (C-C motif) ligand 28 (CCL28), also known as mucous-associated epithelial chemokine (MEC), is a chemokine that regulates the chemotaxis of cells that express the chemokine receptors CCR3 and CCR10^11^,^12^. Several studies indicated that CCL28 was implicated in the angiogenesis of ovarian cancer and rheumatoid arthritis^11^,^13^. However, the molecular mechanism of CCL28 in angiogenesis has not been illustrated clearly.

In the present study, we screened the expression of all chemokines in lung adenocarcinoma (LAC) cell lines under hypoxic condition, producing data of microarray assay and further confirming them by real time RT-PCR. Of all chemokines, only CCL28 was simultaneously up-regulated in lung adenocarcinoma cell lines and tumor samples. Further experiments indicated that CCL28 also plays key roles in tumor angiogenesis by targeting chemokine receptor CCR3 on vascular endothelial cells.

## Materials and Methods

### Cell lines and clinical samples

Lung adenocarcinoma cell lines A549, SPC-A1 and human umbilical vascular endothelial cell line (HUVEC) were purchased from ATCC and maintained in our lab. Human pulmonary microvascular endothelial cell line (HPMEC) was a gift from Simcere Company, which was purchased from Lonza Group Ltd., Switzerland. All the cell lines were cultured in recommended growth medium in 37 °C and 5%CO_2_. Tumor and blood samples were collected from lung adenocarcinoma cancer patients and blood samples from healthy donors were collected as control. Written informed consent was obtained from all subjects before collecting the samples. All the methods were carried out in accordance with the institutional guidelines and approved by the Ethical Review Committee of Jinling Hospital, Nanjing, China.

### Model of hypoxic culture and Microarray analyses

As previously reported^14^, the hypoxic cell culture model was established with hypoxic chambers in our lab. Two lung adenocarcinoma cell lines, A549 and SPC-A1, were cultured under two different oxygen concentrations, 1% and 20%, respectively. The model was confirmed by the expression changes of HIF-1α and its regulated genes such as GLUT1 and VEGFA (Supplementary Figure S1). After culturing for 24 hours, the cells were collected and the total amount of protein and RNA was extracted. Affymetrix GeneChip^®^ Gene 1.0 ST Array System for Human was applied to detect the differences of gene expression of lung adenocarcinoma cell lines cultured under different oxygen concentrations. The experiments were repeated with the same procedures except culturing tumor cell lines in matrigel (BD Bioscience, USA) (3D culture). The fold changes of all chemokine expressions were analyzed independently.

For confirmation of the results, fresh lung adenocarcinoma tumor samples, scissored into pieces with a diameter less than 2 mm, were cultured under two different oxygen concentrations, 1% and 20%, and RNA was extracted for real-time RT-PCR analysis.

### Real-time RT-PCR

Total cellular RNA was extracted from different cell types using TRIzol (Invitrogen, USA). Subsequently, reverse transcription and real-time RT-PCR were performed to determine CCL28 expression levels in the StepOne System (Applied Biosystems, Life tech, USA). Relative gene expression was determined by the ΔΔCt method based on glyceraldehyde-3-phosphate dehydrogenase (GAPDH) levels, and results were expressed as fold change over different conditions.

### Western Blot

Proteins were extracted from the cultured cells by lysis buffer, separated by SDS-PAGE, and subsequently transferred to PVDF membranes (Millipore, USA). The filters were blocked in Tris-buffered saline containing 0.2% Tween plus 5% non-fat milk and incubated with primary antibodies overnight at 4 °C. Specific secondary antibodies were used for detection and visualized through chemiluminescence (ECL, Amersham Pharmacia Biotech, UK). Primary antibodies against HIF-1α, CCR3 (Abcam, USA), p38 MAPK and phosphorylated p38 MAPK (Tyr322) antibodies (SAB, USA), Akt and phosphorylated Akt (Ser473) (Cell Signaling Technology, USA), eNOS and phosphorylated eNOS(Ser1177) (Cell Signaling Technology, USA), PKCα/β II and phosphorylated PKCα/β II(Thr638/641) (Cell Signaling Technology, USA) and GAPDH (Abcam, USA) were included in the present study.

### ELISA

Human CCL28 ELISA kit (Abcam, USA) and VEGF ELISA kit (R&D Systems, USA) were used according to the manufacturer’s instructions to quantify concentrations of CCL28 and VEGF in the serum of lung adenocarcinoma patients, or culture medium of cancer cell lines.

### Angiogenesis assays

Scratch healing rate was applied to analyze the migration of human endothelial cells. Endothelial cells were plated at a seeding density of 5 × 10^4^ cells per well in a 24-well plate and grown until confluent. Standard size wound in the endothelial monolayer was made with a sterile 200 μl micropipette tip. Subsequently, the cells were washed three times with phosphate-buffered saline (PBS). Then complete culture medium containing different concentrations of recombinant human CCL28 (R&D Systems, USA, 0 ng/ml, 1000 ng/ml, 2000 ng/ml) was added to the cells. Photos were taken with a Zeiss inverted microscope at 0 h and again after every 6 hours at the same positions. Cell migration rate was calculated as (wound at 0 h- wound at 24 h)/(wound area at 0 h) × 100.

Ten thousand and 2 × 10^5^ endothelial cells were seeded in 24 well plate pre-coated with matrigel for cell proliferation and tube formation analysis respectively. Recombinant human CCL28 and recombinant human VEGFA (10 ng/ml, R&D Systems, USA) were added into culture medium. To confirm the receptor CCR3 in the function of CCL28 on angiogenesis in lung adenocarcinoma, a neutralizing antibody to CCR3 (CCR3 Ab, monoclonal Rat IgG_2A_ Clone # 61828, 0.5 μg/ml, R&D Systems, USA) was also added into the culture medium. Cell clones and tube numbers were counted under microscopy after culturing for 24 hours and 12 hours, respectively.

Chick chorioallantoic membrane (CAM) and *in vivo* Matrigel assay was carried out for *in vivo* angiogenesis assays. Briefly, Gelatin Sponge blocks containing recombinant human VEGF (10 ng/ml) and recombinant human CCL28 (2000 ng/ml) were applied to the CAM surface of 7-days-old embryos. After 24 hours incubation at 37 °C, the loaded blocks were removed. Before and after the treatment, the CAM surface was photographed at the same position with a digital camera, and the area of newly formed vessels was calculated. Normal saline and cisplatin (2 μg/ml) were used as negative and positive controls.

### Animal models

T cell deficient BALB/c nude mice (CByJ.Cg-*Foxn1*^*nu*^/J) purchased from Model Animal Research Center of Nanjing University were used in the present study. All the animal experiments were carried out in accordance with the institutional guidelines and approved by the Ethical Review Committee of Comparative Medicine, Jinling Hospital, Nanjing, China.

Matrigel (BD Biosciences, USA) containing recombinant human CCL28 (2000 ng/ml) was injected subcutaneously into female BALB/c nude mice (4–6weeks old, 500 μl per mouse). After 7 days, the implanted matrigel was removed with skin and fixed in neutral buffered formalin and embedded in paraffin for immunofluorescence staining with anti-CD31 antibody (Abcam, USA). Neovascularization was recorded using a Zeiss microscope and the area of newly formed vessels was calculated. Normal saline was used as negative control.

Lentivirus vector expressing CCL28 or CCL28 interfering RNA (target sequence, 5′-TCCTGGAAAGAGTGAATAT-3′) was purchased from Lifetech (Shanghai, China) and Genechem (Shanghai, China) company. Lung adenocarcinoma cell lines were infected with an MOI as 1:10 in A549 cells and 1:100 in SPC-A1 cells. Polybrene (Sigma, USA) at the concentration of 8 μg/ml was added to enhance the infection. Blasticidin (0.5 μg/ml) and puromycin (1 μg/ml) were used to screen the stable infected cells. The modulation of CCL28 expression was verified by ELISA or real time RT-PCR.

Cells with CCL28 over expressing or CCL28 knockdown were cultured and implanted subcutaneously in nude mice (female, 4–6 weeks old) with a total cell number of 1 × 10^6^ per mouse. Tumor volumes were recorded every other day and angiogenesis was analyzed by immunohistochemistry.

### Immunohistochemistry, Immunofluorescence and Immunocytochemistry studies

Tumor samples were fixed in 4% formalin and embedded in paraffin. Adjacent 3-mm sections were made for staining. Evaluation of expression of different molecules was independently performed by two experienced pathologists in a blind fashion. Expression intensities of HIF-1α and CCL28 were semi-quantitatively estimated according the immunostaining intensity and positive cell distribution. Briefly, percentages of positive tumor cells determined in at least five areas by 400 magnifications were averaged. The mean percentage was then assigned to five categories: 0, <5; 1, 5 to 25%; 2, 25 to 50%; 3, 50 to 75% and 4, >75%. The intensity of immunostaining was scored as follows: 1, weak; 2, moderate; and 3, intense. For the heterogeneous staining in sections, the predominant pattern was taken into account for scoring. The scores of positive cell percentage and staining intensity were multiplied to produce a weighted score for each case. Immunofluorescence was applied to detect the microvascular density in tumors. Rabbit anti-human/mouse HIF-1α, CCL28, CD34, CD31, CCR3 or CCR10 antibodies (Abcam, USA) were used in immuno-staining.

For immunocytochemistry studies, cells were washed with 1 × PBS and fixed with acetone for 10 minutes on ice. After three washes with 1 × PBS, the cells were incubated with a primary antibody at 4 °C overnight and then treated with the same procedures in immunohistochemistry studies.

### Cell signaling pathway analyzed by Phospho-antibody array

Cell lysates obtained from HPMEC cells (treated with recombinant human VEGFA and recombinant human CCL28, with PBS as a control) were applied to the VEGFR and GPCR-MAPK Pathway Phosphorylation Antibody Array (Full Moon BioSystems, USA), containing 185 and 193 antibodies, respectively. Each of the antibodies has 6 replicates that are printed on standard-size coated glass microscope slides. In brief, The Antibody Array was first blocked with blocking solution (Full Moon BioSystems, USA) for 30 minutes at room temperature, followed by incubation with the biotin-labeled cell lysates at 4 °C overnight. After washing 3 times, the conjugated labeled proteins were detected using Cy3-conjugated streptavidin. For each antibody, phosphorylation ratio was computed as the equation: phosphorylation ratio = (phopho_expeiment_/unphospho_experiment_)/(phopho_control_/unphospho_control_). The results of the Phospho-antibody array were further confirmed by Western Blot assay.

### Photograph and Statistical analyses

The vascular area or formed tube length on photographs were calculated by ImageJ with different plugins (http://imagej.nih.gov/ij/). Data were expressed as mean ± SEM. One-way ANOVA analysis of difference was used for comparisons among multiple groups, followed by Student’s post hoc two-tailed t test. Student’s unpaired two-tailed tests were used for comparisons between two groups. Pearson or Spearman correlation was also applied to analyze the relation between the expression scores of CCL28 and HIF-1α or between the serum levels of CCL28 and VEGFA. SPSS 16.0 was used for all the statistical analyses. p < 0.05 was considered significant.

## Results

### CCL28 expression was up-regulated under hypoxic condition in lung adenocarcinoma cells

**Figure 1:**
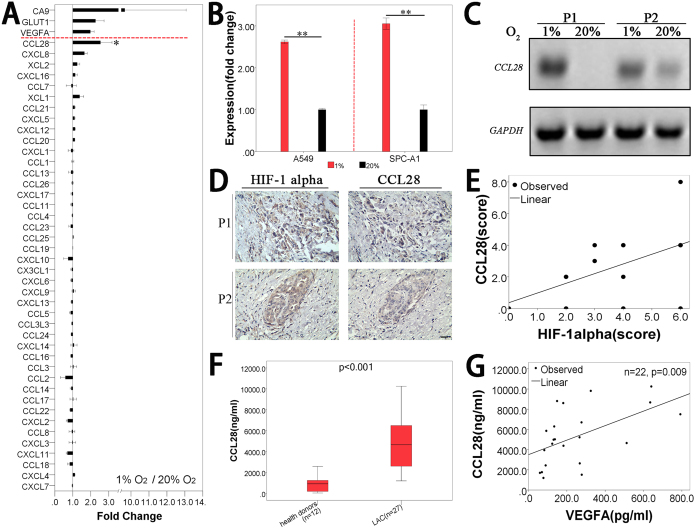
Expression of CCL28 under hypoxic condition in lung adenocarcinoma cells and clinical samples. (**A**) Expression changes of all the chemokines in lung adenocarcinoma cells under hypoxic condition. Lung adenocarcinoma cells, A549 and SPC-A1, were cultured on plates (2D) or in matrigel (3D) under hypoxic condition (1% O_2_) for 24 hours. Cells cultured under normoxia (20% O_2_) were set as control. Several classical hypoxia induced genes, such as *CA9*, *GLUT1* and *VEGFA*, were significantly up-regulated. Of all chemokines, CCL28 was the only one that significantly up-regulated and there was slightly up-regulation of CXCL8. (**B**) Confirming the results from microarray by real time RT-PCR. Expression of CCL28 was significantly up-regulated in both A549 and SPC-A1 cells when cultured in hypoxia chamber (1% O_2_). (**C**) fresh lung adenocarcinoma tumor samples(n = 4), scissored into pieces with a diameter less than 2 mm, were cultured under two different oxygen concentrations, 1 and 20%. RT-PCR was applied to detect the expression of CCL28. Expression of CCL28 was significantly up-regulated in two lung adenocarcinoma tumors (P1 and P2) when cultured in hypoxia chamber (1% O_2_). The bands were cropped from the original gel images in Supplementary Figure S5. (**D,E**) expression of CCL28 in lung adenocarcinoma tumors. Distributions of CCL28 were consistent with that of HIF-1alpha, a standard hypoxia marker (D, representative photos from two patients, P1 and P2). And the intensity of CCL28 expression was also correlated with that of HIF-1alpha expression (**E**), n = 15 and p < 0.05. (**F**) serum concentration of CCL28 was much higher in lung adenocarcinoma patients, comparing with that in healthy donors (p < 0.001). (**G**) as a classical hypoxia induced gene, we further examined the serum concentration of VEGFA in lung adenocarcinoma patients. There was a significant correlation between the serum concentration of CCL28 and VEGFA (n = 22, p = 0.009). Data were expressed as mean ± SEM. LAC, lung adenocarcinoma. **p < 0.01; scale bar, 50 μm.

Tumor hypoxia is a characteristic feature of solid tumors resulting from an imbalance between oxygen (O_2_) supply and consumption. The hypoxic chamber is a widely used model for studies of hypoxia. In the present study, house-made hypoxic chambers with controlled oxygen concentrations were used for the culture of lung adenocarcinoma cells and patients’ tumor tissues. The model was verified by the expressions of hypoxia induced genes, such as *HIF1α*, *GLUT1* and *VEGFA* (Supplementary Figure S1). It seemed that *GLUT1* and *VEGFA* could be induced after 12 hours culture under 1% O_2_, and the effect could be maintained until about 24 hours (Supplementary Figure S1,A). So we kept the hypoxic culture time for 24 hours in the present study. The gene microarray analysis indicated that *CCL28* (fold change, 2.54 ± 0.64) was the only up-regulated one in all the chemokines, which is even higher than *VEGFA* (fold change, 1.98 ± 0.21) (Fig. 1A). The microarray results were confirmed by real time RT-PCR (Fig. 1B). Subsequently, *CCL28* expression was also examined by RT-PCR in lung adenocarcinoma tumor tissues cultured under hypoxic condition (n = 4). Consistent with the results in tumor cell lines, expression of *CCL28* was also highly up-regulated by hypoxia in adenocarcinoma tumor tissues (Fig. 1C).

### CCL28 expression was up-regulated in lung adenocarcinoma patients and related with hypoxia

HIF-1α could be used as a marker of tumor hypoxia. We further explored the relation between CCL28 expression and HIF-1α expression in 15 lung adenocarcinoma tumor samples by immunohistochemistry. Fifteen tumor samples from radical operation were collected (Clinical Stage I 9 and II 6; male 8 and female 7; age 41~79, Supplementary Table 1.). CCL28 was highly expressed in 46.7% (7/15) of the tumor samples. The expression of CCL28 was consistent with the expression of HIF-1α, not only in the spatial distribution (Fig. 1D), but also in the expression intensity (Fig. 1E, Pearson correlation = 0.52, p = 0.047, R square = 0.271). Serum concentration of CCL28 of lung adenocarcinoma patients was further studied. Thirty-nine samples (12 from healthy donors and 27 from lung adenocarcinoma patients) were collected. All of the cancer patients were diagnosed with stage IV disease (male 11 and female 16; age 41~70). The healthy donors were outpatients for conventional physical examination (male 5 and female 7; age 45~62). Interestingly, CCL28 expression was also much higher in the blood serum of lung adenocarcinoma patients, compared to that in the serum of healthy donors (5009.10 ± 504.76ng/ml *vs*. 902.27 ± 221.50 ng/ml, p < 0.001) (Fig. 1F and Supplementary Table 2). As VEGFA is one of the hypoxia induced genes, the concentration of VEGFA in the serum of lung adenocarcinoma patients was examined by ELISA (22 in 27 blood samples for the study of serum concentration of CCL28). There was a very significant correlation between the concentration of CCL28 and that of VEGFA in the serum of adenocarcinoma patients (Fig. 1G, Pearson correlation = 0.54, p = 0.009, R square = 0.291).

### CCL28 receptor, CCR3 but not CCR10, was highly expressed in the vascular endothelial cell in lung adenocarcinoma

**Figure 2:**
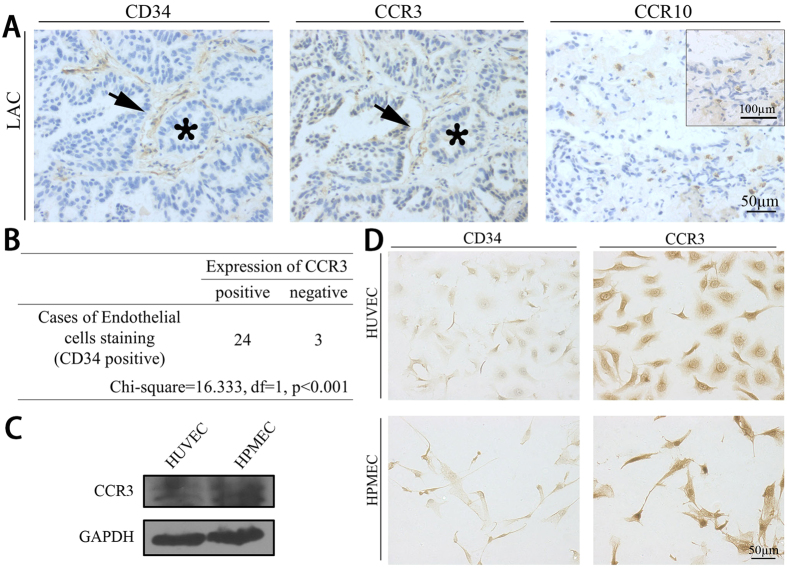
Expression of the CCL28 receptors, CCR3 and CCR10, in lung adenocarcinoma tumors and vascular endothelial cells. (**A**) representative photos for CD34, CCR3 and CCR10 expressions in lung adenocarcinoma tumors examined by immunohistochemistry. The distribution of CCR3 was consistent with that of CD34, a widely used vascular endothelial cell marker (Left and medium panels, black stars indicating the same position in the sections of lung adenocarcinoma tumors and black arrows indicating the same distribution of CD34 and CCR3 expression). While expression of CCR10 was mostly distributed in infiltrated inflammatory cell in lung adenocarcinoma tumors (right panel and magnification in right corner). (**B**) Distribution of CCR3 was consistent with that of CD34 in tumor sample (24 in 27, p < 0.001). (**C**) Expression of CCR3 in HUVEC and HPMEC was examined by Western Blot. The bands were cropped from the original blot images in Supplementary Figure S5. (**D**) Expressions of CD34 and CCR3 in human umbilical vascular endothelial cell (HUVEC) and human pulmonary microvascular endothelial cell (HPMEC). CCR3 was highly expressed in the two cells (right panels). Scale bar, 50 μm.

CCR3 and CCR10 are both receptors of CCL28. We analyzed adjacent sections of paraffin embedded lung adenocarcinoma samples (n = 27) by immunohistochemistry with antibodies against CD34, CCR3 and CCR10. CD34 is a widely used marker for vascular endothelial cells. As indicated in Fig. 2A,B, the distribution of CCR3 expression was consistent with that of CD34 expression (Chi-square = 16.3333, df = 1, p < 0.001). However, CCR10 was mostly expressed on the inflammatory cells in tumor stroma (Fig. 2A, right panel). Subsequently, expression of the two receptors was also detected in vascular endothelial cell lines, HUVEC and HPMEC. Similar to the results from tumor samples, CCR3 was highly expressed on both of the two cell lines (Fig. 2D). CCR10 was also expressed on HUVEC cells, but not on HPMEC cells (Supplementary Figure S2). Furthermore, the expression of CCR3 on HUVEC and HPMEC was further confirmed by Western Blot (Fig. 2C). Taken together, the evidences indicated that CCR3, but not CCR10, was the main receptor of CCL28 on the microvascular endothelial cells, especially in lung adenocarcinoma.

### CCL28 promoted angiogenesis *in vitro*

**Figure 3:**
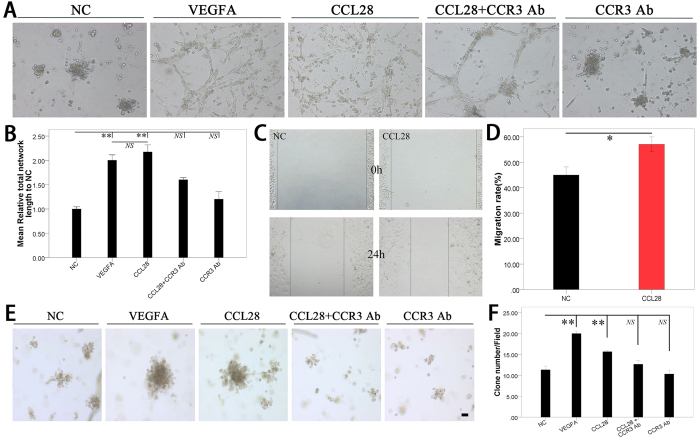
CCL28 promoted tube formation, migration and proliferation of endothelial cells. **(A,B**) human pulmonary microvascular endothelial cells (HPMEC) were seeded in 24-well plate pre-coated with matrigel (5 × 10^4^/well). The total tube length was analyzed by ImageJ. CCL28 (2000 ng/ml) and VEGFA (10 ng/ml) significantly increased the formed tube length of HPMEC. In addition, the effect of CCL28 on tube formation was neutralized by antibody against CCR3 (CCR3 Ab). (**C,D**) wound healing assay of HUVEC indicated that CCL28 increased migration of endothelial cells. (**E,F**) when seeded with a low number (1 × 10^4^cells/well) on matrigel, HPMEC cells could proliferate and form cell clones. The number of cell clones was significantly increased by CCL28 (p < 0.01), as well as VEGFA, and this effect of CCL28 could also be neutralized by neutralizing CCR3 antibody. NC, negative control; NS, no significant. Data were expressed as mean ± SEM. *p < 0.05; **p < 0.01; scale bar, 50 μm.

In the tube formation assay, CCL28 could increase the total tube length of both HUVEC and HPMEC in a dose dependent manner, nearly the same as VEGFA (10 ng/ml) at a concentration of 2000 ng/ml (Fig. 3A and Supplementary Figure S3), so we fixed the concentration of 2000 ng/ml for all the following tests of CCL28. In addition, the effects on vascular endothelial cell tube formation of CCL28 could be inhibited by a neutralizing antibody against CCR3 (CCR3 Ab), which could not induce angiogenesis alone (Fig. 3A,B). Migration of vascular endothelial cells could also be enhanced by CCL28 (Fig. 3C,D). When seeded with a low number on matrigel, HPMEC cells could proliferate and form cell clones. The number of cell clones was significantly increased by CCL28 (p < 0.01), as well as VEGFA. This effect could also be neutralized by CCR3 antibody (Fig. 3E,F).

### CCL28 promoted angiogenesis *in vivo*

**Figure 4:**
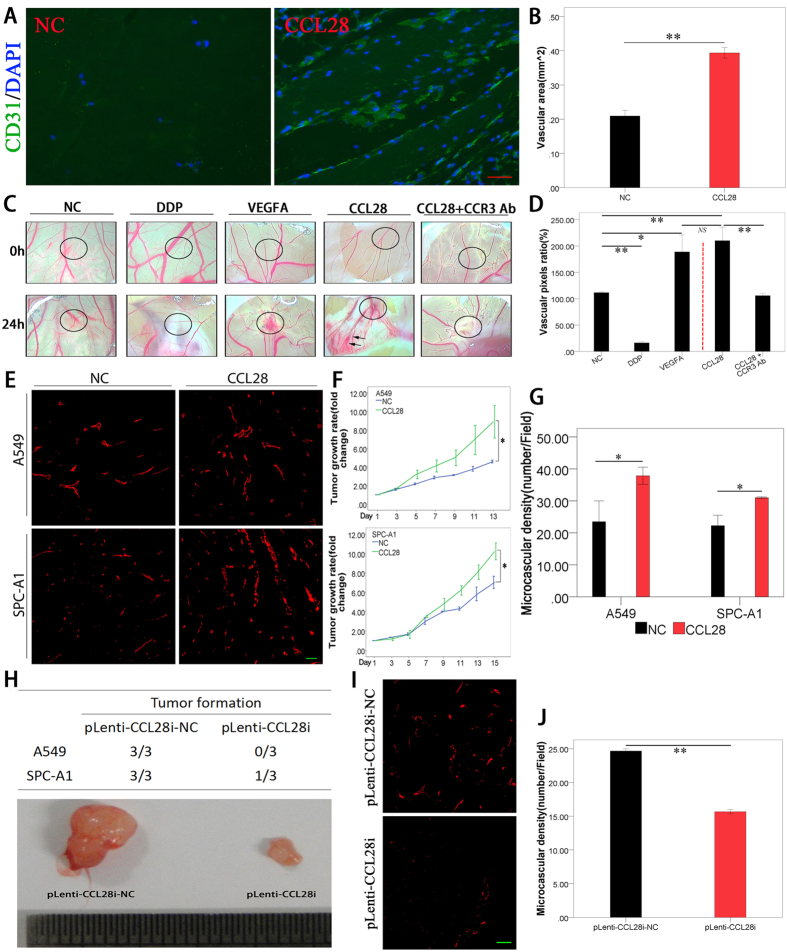
CCL28 promoted angiogenesis *in vivo*. (**A,B**), matrigel was mixed with CCL28 (2000 ng/ml) and implanted into T cell deficient BALB/c nude mice (CByJ.Cg-*Foxn1*^*nu*^/J, female, 4–6 weeks old) subcutaneously. CCL28 induced more angiogenesis in matrigel, which was analyzed by immunofluorescence and the vascular area was calculated (**B–D**), effects of CCL28 on angiogenesis analyzed by Chick chorioallantoic membrane (CAM) assay. As a positive and negative control, VEGFA (10 ng/ml) and cisplatin (DDP, 2 μg/ml) could significantly induce and inhibit angiogenesis on chick chorioallantoic membrane, respectively (p < 0.01). While CCL28 (2000 ng/ml) also induced more angiogenesis on chick chorioallantoic membrane. Furthermore, the effect of CCL28 on angiogenesis was neutralized by antibody against CCR3 (CCR3 Ab). Black circles indicated the positions where the Gelatin Sponges were placed. Vascular areas were analyzed on the whole taken photos. (**E–G**), lung adenocarcinoma cell lines, A549 and SPC-A1, with stable over-expression of CCL28 were screened out and implanted subcutaneously in BALB/c nude mice. Compared with control, tumors with CCL28 high expression had a significant higher growth rate (**F**, p < 0.05). In addition, the tumor microvascular density was much higher in CCL28 high expression tumors (**E** and **G**, p < 0.05). (**H–J**), A549 and SPC-A1 cells with CCL28 expression knockdown by RNA interfering had a lower rate of tumor formation in BALB/c nude mice (**H**). And there was a lower level of tumor microvascular density in CCL28 knockdown tumors (**I** and **J**, p < 0.05). The vascular areas or densities were analyzed by ImageJ. NC, negative control; NS, no significant. Data were expressed as mean ± SEM. *p < 0.05; **p < 0.01; scale bar, 50 μm.

Similar to the *in vitro* studies, angiogenesis was also promoted by CCL28 *in vivo*. CCL28 induced more angiogenesis when mixed in matrigel and implanted into BALB/c nude mice subcutaneously, which was analyzed by immunofluorescence (Fig. 4A,B).

As a positive and negative control, VEGFA (10 ng/ml) and cisplatin (2 μg/ml) could significantly induce and inhibit angiogenesis on chick chorioallantoic membrane, respectively (Fig. 4C,D, p < 0.01). CCL28 also promoted angiogenesis on the chick chorioallantoic membrane (p < 0.01, compared with control, Fig. 4C,D).

Lung adenocarcinoma cell lines, A549 and SPC-A1, with stable over expression of CCL28 were screened out and implanted subcutaneously in BALB/c nude mice. Compared to the control, tumors with CCL28 high expression had a significant higher growth rate (n = 5–6, Fig. 4F, p < 0.05). In addition, the tumor microvascular density was much higher in CCL28 high expression tumors (Fig. 4E,G, p < 0.05). Reversely, A549 and SPC-A1 cells, with CCL28 expression knock down had a lower rate of tumor formation in BALB/c nude mice (n = 3, repeated twice, Fig. 4H) and there was a lower level of tumor microvascular density in CCL28 knockdown tumors (Fig. 4I,J, p < 0.05). The animal experiment was repeated twice with similar results (Supplementary Figure S3,E).

### CCL28 activated CCR3 and bypass the VEGFR signaling in microvascular endothelial cell

**Figure 5:**
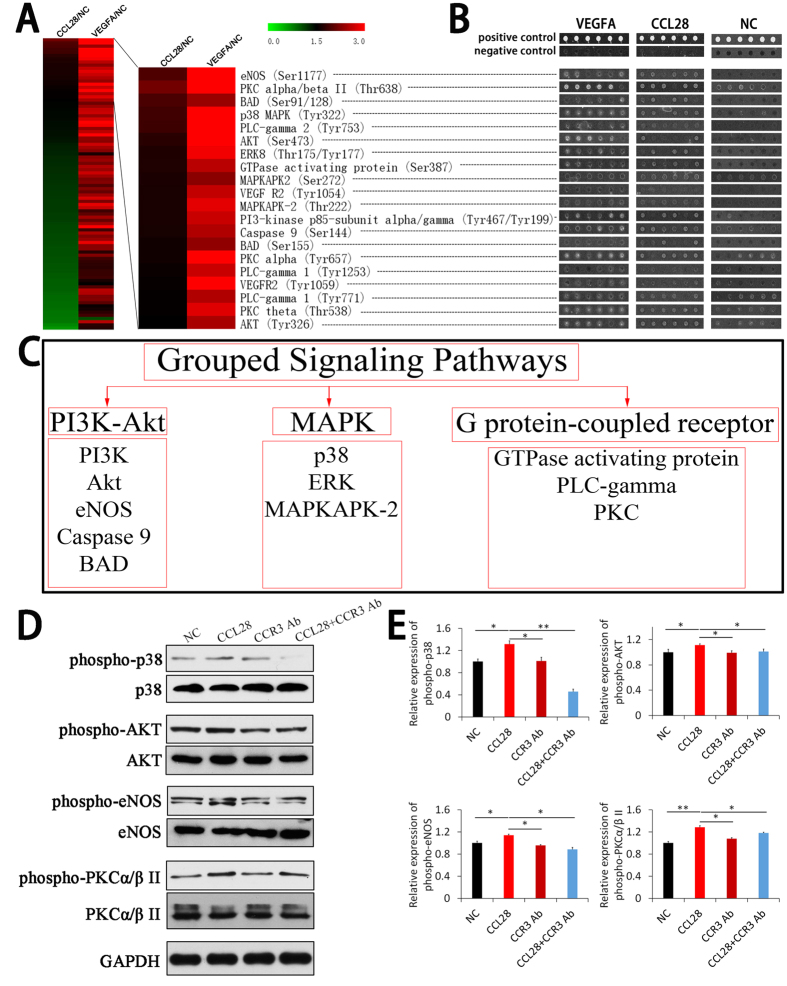
Cell signaling pathways activated by CCL28 in HPMEC. Phosphorylation Antibody Arrays were applied to detect the changes of phosphorylation of signaling proteins in HPMEC cells treated by CCL28 (2000 ng/ml) and VEGFA (10 ng/ml), with normal saline as control. The cut-off value for the phosphorylation was set up as 1.5. (**A**) heat map represented the results of VEGFR Pathway Phosphorylation Antibody Array. As a positive control, VEGFA (10 ng/ml) activated the whole VEGFR signaling pathway (**A**, left panel). CCL28 also induced phosphorylation of twenty signaling proteins (**A**, right panel). (**B**) scanned photos of Phosphorylation Antibody Arrays, consistent with twenty phosphorylated signaling proteins in A (right panel) as indicated by dashed lines. (**C**) the phosphorylated proteins induced by CCL28 could be grouped into three signaling pathways, including PI3K-Akt, MAPK and G protein-coupled receptor signaling pathways. (**D,E**) Verification of the results of Phosphorylation Antibody Arrays. Western Blot was applied to detect phosphorylation of p38 MAPK (Tyr322), Akt (Ser473), eNOS(Ser1177) and PKCα/β II(Thr638/641) in HPMEC cells treated with CCL28 (2000 ng/ml). The bands were cropped from the original blot images in Supplementary Figure S5. p38 MAPK, Akt, eNOS and PKCα/β II were significantly phosphorylated in HPMEC cells after treatment of CCL28. And this effect was neutralized by antibody to CCR3 (CCR3 Ab). NC, negative control. Data were expressed as mean ± SEM. *p < 0.05, **p < 0.01.

**Figure 6:**
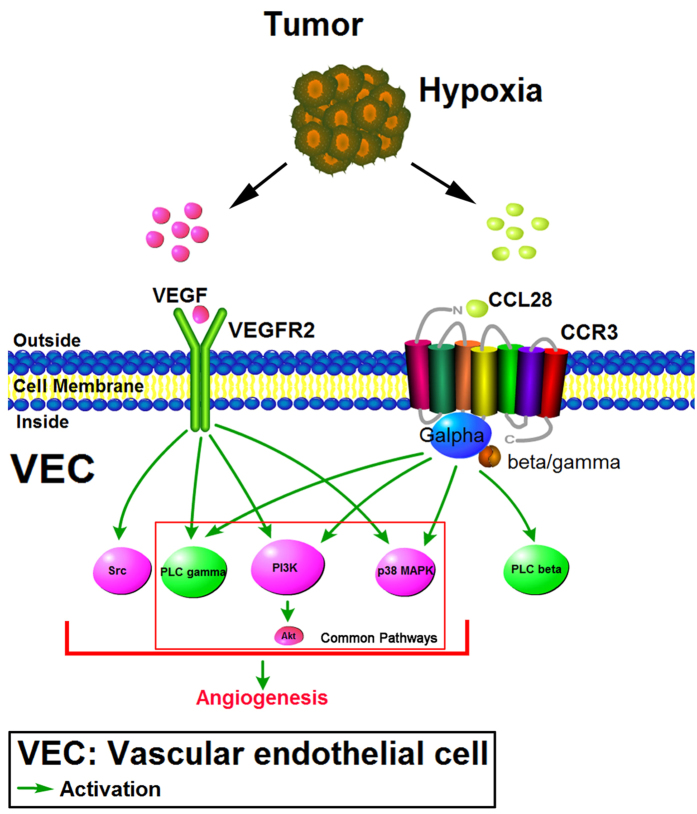
Diagram of the activation of CCL28/CCR3 signaling pathways in vascular endothelial cells. Facing the stress of hypoxia, tumor cells up-regulate the expressions of CCL8 and VEGF, both of which could modulate functions of vascular endothelial cells through directly activating their receptors, CCR3 and VEGFR, on the cells. There are three common pathways between CCL28/CCR3 and VEGFA-VEGFR2 signaling (on the levels of PI3K-Akt, p38 MAPK and PLC gamma). As a result, CCL28 could promote angiogenesis in lung adenocarcinoma and bypass the effects of VEGFA.

To figure out the whole effect of CCL28 on microvascular endothelial cell signaling, Phosphorylation Antibody Arrays were applied to detect the changes of phosphorylation of signaling proteins in HPMEC cells. The cut-off value for the phosphorylation was set up as 1.5. As a positive control, VEGFA (10 ng/ml) could activate the whole VEGFR signaling pathway (Fig. 5A, left panels and B) and most of the GPCR-MAPK signaling pathway (Supplementary Figure S4,A, left panels and B). Although with a lower intensity, CCL28 also induced phosphorylation of twenty signaling proteins, which could be grouped into three signaling pathways, including PI3K-Akt, MAPK and G protein-coupled receptor signaling pathways (Fig. 5C and Supplementary Figure S4,C). The results were verified by Western Blot. Phosphorylations of p38 MAPK, Akt, eNOS and PKC were induced by CCL28 in HPMEC cells, and the phosphorylate effects could be inhibited by CCR3 antibody (Fig. 5D,E). Taken together, activation of CCL28-CCR3 and VEGFA-VEGFR shared three common pathways on the level of PI3K-Akt, p38 MAPK and PLC gamma (Fig. 6). As a result, CCL28 could promote angiogenesis in lung adenocarcinoma and bypass the effects of VEGFA. Interestingly, VEGFR2 was also phosphorylated after treatment of CCL28, at the positions of Tyr1054 and Tyr 1059, indicating that there might be a crosstalk on the receptor level which needed to be further studied.

## Discussion

Hypoxia is a feature of most tumors, and influences many aspects of the biology of tumors, such as angiogenesis, invasiveness, metastases, immunosuppression, drug resistance, altered metabolism and genomic instability^15^. Previous clinical studies have proved that hypoxia is a prognostic factor in many tumors, including lung adenocarcinoma^16^.

Human lung adenocarcinoma is a common histological form of lung cancer that contains certain distinct malignant tissue architectural, cytological, or molecular features, and tends to form metastases widely at an early stage^17^. Hematogenous metastasis of lung adenocarcinoma is related to the tumor angiogenesis^18^. Some antiangiogenesis drugs have been approved for clinical application in advanced lung adenocarcinoma patients^19^. However, the effects of these drugs may only improve the progression free survival (PFS) of patients. The fact that antigangiogenesis therapy may promote the progress of malignant tumors becomes a major concern^5^,^6^. Discrepancies between pre-clinical and clinical results indicate that the mechanism of angiogenesis should be fully illustrated before applying antiangiogenesis therapies in patients^20^.

A large amount of data supports that hypoxia drives tumor angiogenesis^21^. Hypoxia promotes vessel growth by up-regulating multiple pro-angiogenic pathways that modulate many aspects in tumor vascular biology. The transcription factor hypoxia-inducible factor-1 (HIF-1) and genes induced by HIF-1 might be key regulators responsible for the induction of tumor angiogenesis^22^. In the present study, expressions of a lot of genes (more than 1 700, including that control tumor angiogenesis, metabolism and cell cycle) were changed in lung adenocarcinoma cells after exposure under hypoxic condition (1% O_2_). Although the molecular mechanism was not so clear, few studies indicated that expressions of some chemokines, such as CXCL8 and CCL2, were induced by hypoxia^23^,^24^. However, consistent with that in ovarian cancer^13^, only expression of CCL28 was up-regulated in lung adenocarcinoma cells when the cells were cultured under hypoxic condition.

Several chemokines, such as CXCL12/SDF-1, CXCL8 and CCL2, have been reported as pro-angiogenesis factors in different solid tumors^25^,^26^,^27^,^28^. The molecular mechanisms were mostly ascribed to high expression of VEGF from tumor cells or tumor stromal cells recruited by these chemokines. Very few studies focus on the direct effect of these chemokines on the vascular endothelial cells. There are even less reports on the pro-angiogenesis chemokines in lung adenocarcinoma. However, evidences supporting a direct role of CCL28 on tumor angiogenesis were presented in the present study.

The receptors of CCL28 are CCR3 and CCR10, both of which are G protein-coupled receptors (GPCRs). CCR3 is highly expressed in eosinophils, and is also detected in Th1 and Th2 cells, as well as in airway epithelial cells. This receptor may contribute to the accumulation and activation of eosinophils and other inflammatory cells in the allergic airway, and possibly at sites of parasitic infection. CCR3 could also bind and respond to a variety of other chemokines, including eotaxin (CCL11), eotaxin-3 (CCL26), MCP-3 (CCL7), MCP-4 (CCL13), and RANTES (CCL5)^29^, whereas CCR10 is normally expressed in melanocytes, plasma cells and skin-homing T cells and its ligands are CCL27 and CCL28^30^. Previous studies have proven that CCL28 could recruit CCR3+ regulatory T cells (Tregs) homing to the tumor, which thereafter could promote tumor immunosuppression and tumor angiogenesis by secreting VEGF^13^. However, in the present study, the expression of CCR3 was identified in the microvascular endothelial cells of lung adenocarcinoma, which could be directly affected by CCL28. To minimize the indirect effects of CCL28 on tumor angiogenesis, T cell deficient BALB/c nude mice were used for *in vivo* studies.

Taken together, at least two major roles are played by CCL28 in lung adenocarcinoma, including immunosuppression and pro-angiogenesis (directly and indirectly), both of which are important targets for cancer therapy. Recently, some studies have proven that combination of antiangiogenesis therapy with immunotherapy could have better antitumor effects in lung adenocarcinoma, indicating tumor immunosuppression and angiogenesis might be tightly connected^31^,^32^,^33^,^34^. In conclusion, hypoxia of lung adenocarcinoma cells could induce both tumor immunosuppression and angiogenesis through the up-regulation of CCL28. As a result, CCL28 might be an ideal target to both inhibit tumor immunosuppression and angiogenesis in lung adenocarcinoma.

Meanwhile, although not as effective as VEGFA, activation of CCR3 on microvascular endothelial cells could cause trans-activation of VEGFR2 signaling pathway, and phosphorylate downstream PI3K-Akt, p38 MAPK, PLCγ and even on the level of VEGFR2. Recent studies have shown that signal transduction initiated by GPCRs and receptor tyrosine kinases (RTKs, such as EGFR and VEGFR) is not organized in distinct signaling cassettes where receptor activation causes subsequent effects in a linear manner^35^,^36^. In fact, signal integration arises from a complex network involving “crosstalk” between separate signaling units. Just as indicated in the present study, there are three common pathways between the CCR3 signaling and VEGFR2 signaling. Most importantly, consistent with previous studies^37^, activation of CCR3 could directly cause phosphorylation of VEGFR2. However, the mechanism of this trans-activation is still not clear and needs to be further studied.

In conclusion, CCL28, one of the CC chemokines, is identified as another hypoxia induced molecule in lung adenocarcinoma. Besides other effects on tumor biology, such as immunosuppression, CCL28 could promote angiogenesis in lung adenocarcinoma by directly activating its receptor, CCR3, on microvascular endothelial cells. Furthermore, the signaling pathway after activation of CCR3 could bypass the VEGFR2 signaling pathway in microvascular endothelial cells, which might be the basis of the pro-angiogenesis function of CCL28.

## Additional Information

**Accession code**: The ArrayExpress accession number for the gene expression data reported in the present study is E-MTAB-3512.

**How to cite this article**: Huang, G. *et al.* Hypoxia induced CCL28 promotes angiogenesis in lung adenocarcinoma by targeting CCR3 on endothelial cells. *Sci. Rep.* **6**, 27152; doi: 10.1038/srep27152 (2016).

## Supplementary Material

Supplementary Information
