# Supplementary material for: Hypoxia induced CCL28 promotes angiogenesis in lung adenocarcinoma by targeting CCR3 on endothelial cells
Source: Sci Rep. 2016 Jun 2;6:27152. doi: 10.1038/srep27152 (PMC4890017; doi:10.1038/srep27152)
Supplement: Supplementary Information [file srep27152-s1.pdf]

# Supplementary files for

## Hypoxia induced CCL28 promotes angiogenesis in lung adenocarcinoma by targeting CCR3 on endothelial cells

Guichun Huang[1, #], Leilei Tao[1, #], Sunan Shen[2], Longbang Chen[1, *]

[1]Medical Oncology Department of Jinling Hospital, Medical School of Nanjing University; [2]Medical School of Nanjing University

[#]Authors contribute equally in the work.

***Corresponding to Longbang Chen, dr.chenlb@nju.edu.cn, Tel: 08680600153, 305# Zhongshan Road, Nanjing, China.**

# Supplementary Figure Legends

**Figure S1**. Verification of hypoxic culture model. A, lung adenocarcinoma cells (A549 and SPC-A1) were cultured in the house-made hypoxic chamber with different concentrations of oxygen (1% and 20%). Total RNA was extracted and mixed as different pools under different culture condition. RT-PCR was applied to examine the expression of classic hypoxia induced genes (*VEGFA* and *GLUT1*) with *GAPDH* as internal reference. The culture time was controlled as 12 hours, 24 hours and 48 hours. It seemed that *GLUT1* and *VEGFA* could be induced after 12 hours culture under 1% $O_2$, and the effect could be maintained until for 24 hours. B, further verification of the same gene expressions in different cells, HBE (Human bronchial epithelial cells) and lung adenocarcinoma cells (A549 and SPC-A1). Three cell lines cultured under hypoxic condition showed similar up-regulation in *VEGFA* and *GLUT1*expression. C, Expression of HIF-1α was examined in lung adenocarcinoma cells (A549 and SPC-A1) by Western Blot. The bands were cropped from the original blot images in Supplementary **Figure S5**. Hypoxic culture in the present study induced significant high expression of HIF-1α in A549 and SPC-A1 cells.

**Figure S2.** Expressions of CCR10 in human umbilical vascular endothelial cell (HUVEC) and human pulmonary microvascular endothelial cell (HPMEC). CCR10 expression was relatively weaker in HUVEC (A) and negative in HPMEC (B). Scale bar, 50μm.

**Figure S3.** *In vitro* and *In vivo* angiogenesis effects of CCL28. A-D, Tube formation of different endothelial cells induced by gradient concentrations of CCL28. CCL28 could increase the formed tube length of both HPMEC (A and B) and HUVEC (C and D) in a dose dependent manner. E, Repeated animal experiments with similar results. Lung adenocarcinoma cells with CCL28 expression knockdown (pLenti-CCL28i) by RNA interfering grew slower in BALB/c nude mice. NC, negative control. Data were expressed as mean ± SEM. NS, no significant. **, $p<0.01$.

**Figure S4.** GPCR-MAPK and VEGFR2 signaling pathways activated by CCL28 in HPMEC. A, heat map represented the results of GPCR-MAPK Pathway Phosphorylation Antibody Array. Most proteins of the GPCR-MAPK signaling pathway were activated by VEGFA (10ng/ml) (left panels). Consistent with results in VEGFR Pathway Phosphorylation Antibody Array, PI3K signaling was activated by CCL28 (right panel). B, the photographs of scanned Phosphorylation Antibody Array, consistent with six phosphorylated signaling proteins in A (right panel) as indicated by dashed lines. C, Scheme of the VEGFR2 signaling activated by CCL28 (2000ng/ml). Red and dark green represent high and low phosphorylation of signaling protein, respectively. PI3K-Akt, p38 MAPK and PLC gamma signaling pathway were activated by CCL28. NC, negative control.

**Figure S5.** Original gels and blots of Figure 1C, Figure 2C, Figure 5D and Figure S1C. DNA products were detected by agarose gel electrophoresis. And the blots were scanned from the X-ray films. The cropped bands were framed with black lines.

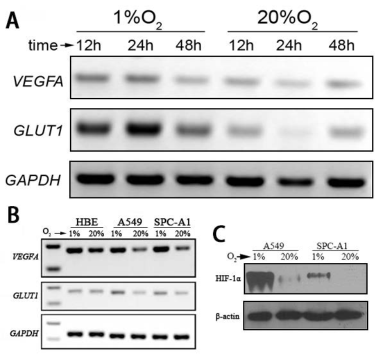
A
1%O2
20%O2
time
12h
24h
48h
12h
24h
48h
VEGFA
GLUT1
GAPDH
B
O2
HBE
A549
SPC-A1
1%
20%
1%
20%
1%
20%
VEGFA
GLUT1
GAPDH
C
A549
SPC-A1
O2
1%
20%
1%
20%
HIF-1α
β-actin

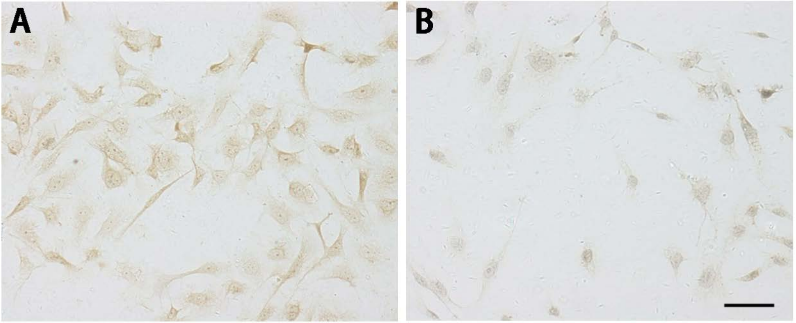
A
B

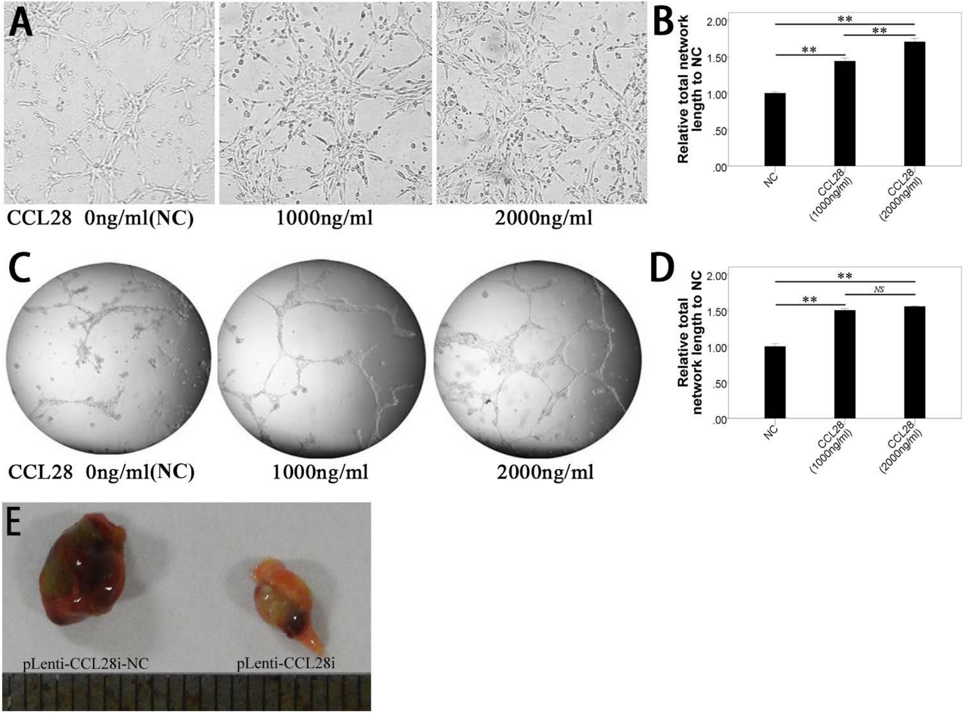

A
CCL28 0ng/ml(NC)
1000ng/ml
2000ng/ml
B
Relative total network length to NC
2.00
1.50
1.00
.50
.00
NC
CCL28 (1000ng/ml)
CCL28 (2000ng/ml)
**
**
**
C
CCL28 0ng/ml(NC)
1000ng/ml
2000ng/ml
D
Relative total network length to NC
2.00
1.50
1.00
.50
.00
NC
CCL28 (1000ng/ml)
CCL28 (2000ng/ml)
**
NS
**
E
pLenti-CCL28i-NC
pLenti-CCL28i

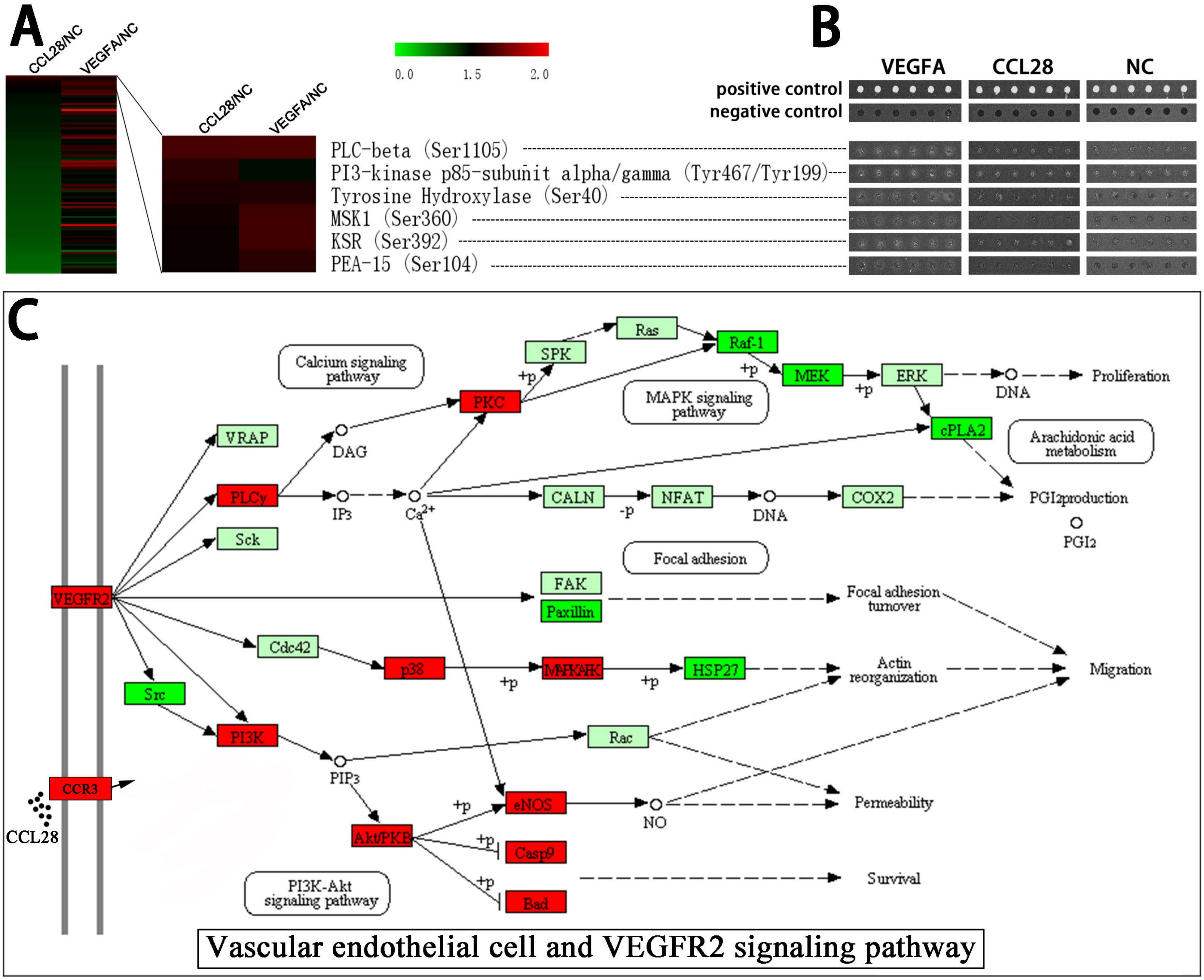
A
CCL28/NC
VEGFA/NC
0.0
1.5
2.0
B
VEGFA
CCL28
NC
positive control
negative control
PLC-beta (Ser1105)
PI3-kinase p85-subunit alpha/gamma (Tyr467/Tyr199)
Tyrosine Hydroxylase (Ser40)
MSK1 (Ser360)
KSR (Ser392)
PEA-15 (Ser104)
C
Calcium signaling pathway
MAPK signaling pathway
Arachidonic acid metabolism
Focal adhesion
PI3K-Akt signaling pathway
SPK
Ras
Raf-1
MEK
ERK
PKC
VRAP
DAG
PLCγ
IP3
Ca2+
CALN
NFAT
COX2
cPLA2
DNA
Proliferation
PGI2production
PGI2
Sck
VEGFR2
FAK
Paxillin
Focal adhesion turnover
Cdc42
p38
MAPKAPK
HSP27
Actin reorganization
Migration
Src
PI3K
Rac
PIP3
CCR3
CCL28
eNOS
NO
Permeability
Akt/PKB
Casp9
Bad
Survival
+p
-p
Vascular endothelial cell and VEGFR2 signaling pathway

# Figure 1C

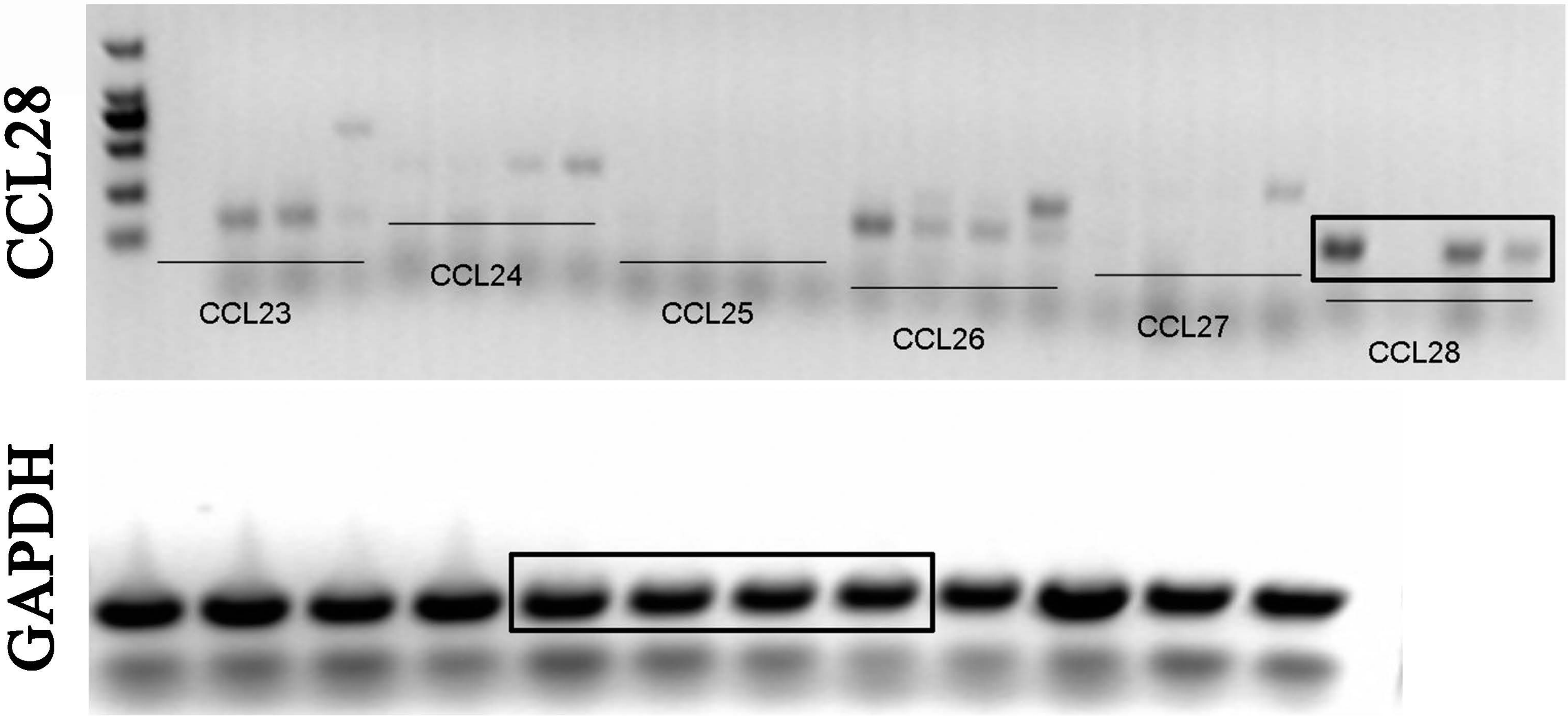


# Figure 2C

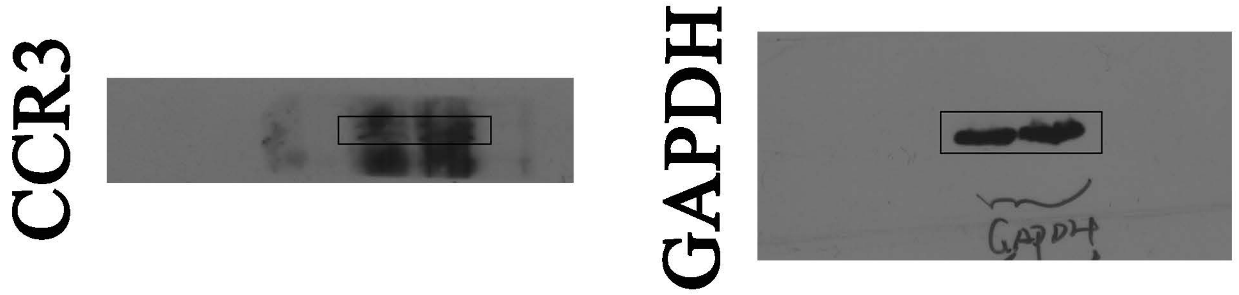


# Figure 5D

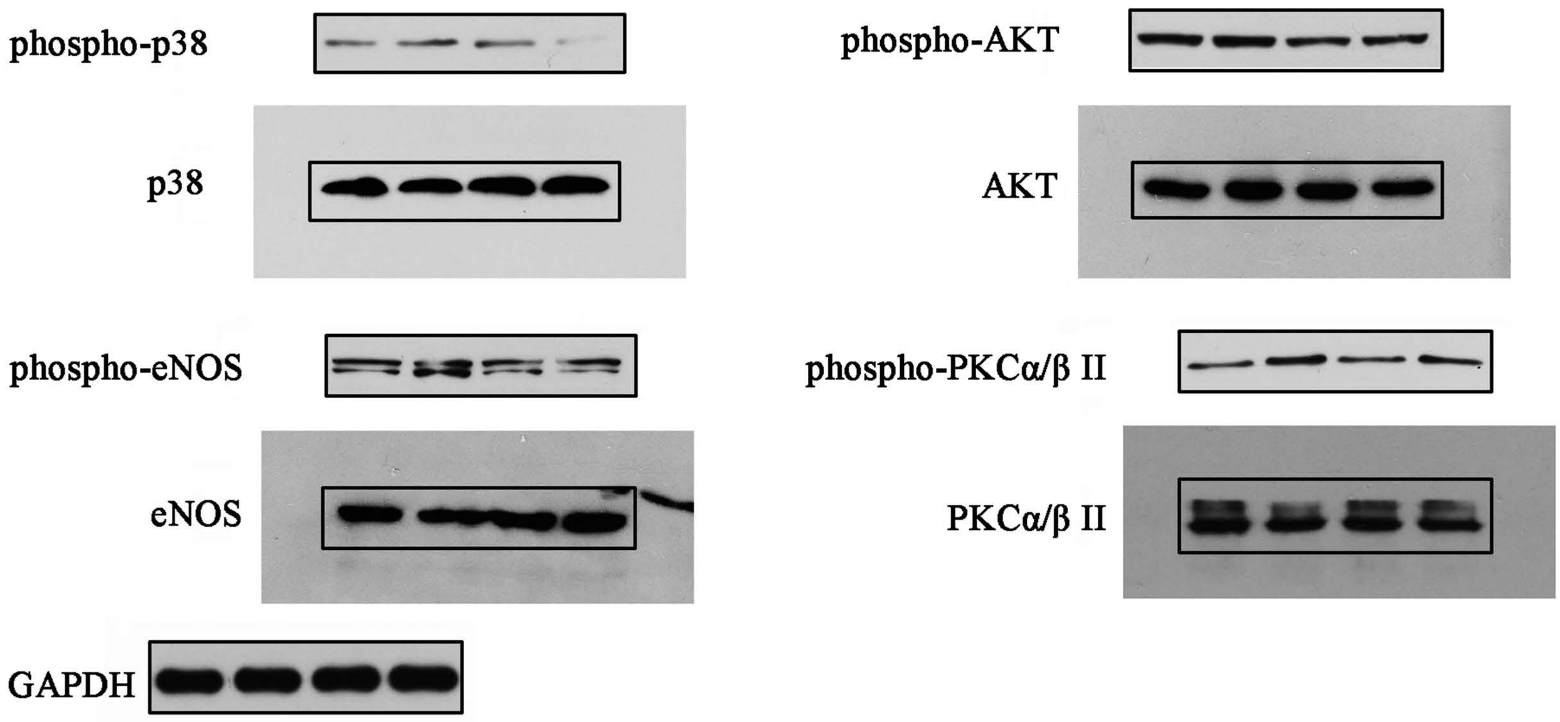


# Figure S1C

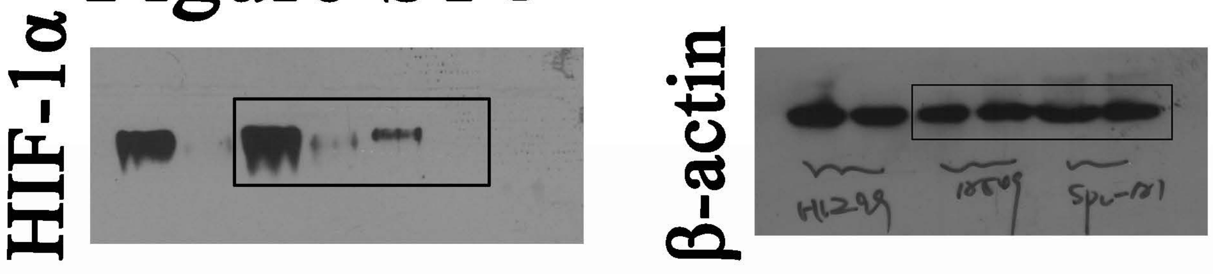

Supplementary Table 1. Characteristics of lung adenocarcinoma patients (LAC) for immunohistochemistry.

| patient no. | sex | age | sample | pathology | stage | Immunostaining |
|---|---|---|---|---|---|---|
| 1 | male | 70 | resected tumor | LAC | I | CCL28/HIF-1α/CCR3 |
| 2 | male | 56 | resected tumor | LAC | II | CCL28/HIF-1α/CCR3 |
| 3 | female | 50 | resected tumor | LAC | I | CCL28/HIF-1α/CCR3 |
| 4 | male | 45 | resected tumor | LAC | II | CCL28/HIF-1α/CCR3 |
| 5 | female | 79 | resected tumor | LAC | II | CCL28/HIF-1α/CCR3 |
| 6 | female | 65 | resected tumor | LAC | I | CCL28/HIF-1α/CCR3 |
| 7 | male | 57 | resected tumor | LAC | I | CCL28/HIF-1α/CCR3 |
| 8 | female | 61 | resected tumor | LAC | I | CCL28/HIF-1α/CCR3 |
| 9 | female | 68 | resected tumor | LAC | I | CCL28/HIF-1α/CCR3 |
| 10 | female | 78 | resected tumor | LAC | I | CCL28/HIF-1α/CCR3 |
| 11 | male | 42 | resected tumor | LAC | II | CCL28/HIF-1α/CCR3 |
| 12 | male | 75 | resected tumor | LAC | I | CCL28/HIF-1α/CCR3 |
| 13 | male | 64 | resected tumor | LAC | II | CCL28/HIF-1α/CCR3 |
| 14 | female | 59 | resected tumor | LAC | I | CCL28/HIF-1α/CCR3 |
| 15 | male | 41 | resected tumor | LAC | II | CCL28/HIF-1α/CCR3 |
| 16 | male | 72 | biopsy | LAC | IV | CCR3 |
| 17 | male | 74 | biopsy | LAC | IV | CCR3 |
| 18 | male | 74 | biopsy | LAC | III | CCR3 |
| 19 | female | 63 | biopsy | LAC | IV | CCR3 |
| 20 | female | 51 | biopsy | LAC | IV | CCR3 |
| 21 | female | 56 | biopsy | LAC | III | CCR3 |
| 22 | male | 70 | biopsy | LAC | IV | CCR3 |
| 23 | female | 59 | biopsy | LAC | IV | CCR3 |
| 24 | male | 52 | biopsy | LAC | IV | CCR3 |
| 25 | female | 47 | biopsy | LAC | III | CCR3 |
| 26 | female | 56 | biopsy | LAC | IV | CCR3 |
| 27 | male | 68 | biopsy | LAC | IV | CCR3 |

Supplementary Table 2. Characteristics of lung adenocarcinoma patients (LAC) for ELISA.

| patient no. | sex | age | pathology | stage |
|---|---|---|---|---|
| 1 | female | 70 | LAC | IV |
| 2 | female | 69 | LAC | IV |
| 3 | male | 64 | LAC | IV |
| 4 | female | 56 | LAC | IV |
| 5 | male | 54 | LAC | IV |
| 6 | female | 41 | LAC | IV |
| 7 | female | 57 | LAC | IV |
| 8 | male | 64 | LAC | IV |
| 9 | male | 45 | LAC | IV |
| 10 | female | 44 | LAC | IV |
| 11 | female | 60 | LAC | IV |
| 12 | male | 53 | LAC | IV |
| 13 | female | 64 | LAC | IV |
| 14 | female | 50 | LAC | IV |
| 15 | male | 64 | LAC | IV |
| 16 | female | 66 | LAC | IV |
| 17 | female | 72 | LAC | IV |
| 18 | male | 61 | LAC | IV |
| 19 | male | 72 | LAC | IV |
| 20 | female | 64 | LAC | IV |
| 21 | female | 56 | LAC | IV |
| 22 | female | 35 | LAC | IV |
| 23 | male | 56 | LAC | IV |
| 24 | female | 51 | LAC | IV |
| 25 | male | 55 | LAC | IV |
| 26 | female | 49 | LAC | IV |
| 27 | male | 49 | LAC | IV |
